# Effects of harvest maturity coupled with various drying methods on the quality and aroma composition of gray jujube powder

**DOI:** 10.1016/j.fochx.2025.102916

**Published:** 2025-08-20

**Authors:** Zhanxia Liu, Hongbin Wu, Yinglin Du, Xinwen Jin, Tarun Belwal, Hui Yang, Xizhe Fu

**Affiliations:** a Institute of Agricultural Products Processing, Xinjiang Academy of Agricultural Reclamation Sciences, Shihezi, Xinjiang 832000, China,; b Inspection Center of the Agricultural Quality and Safety in Urumqi, Urumqi, Xinjiang 830000, China,; c Key Laboratory of Processing and Quality Safety Control of Characteristic Agricultural Products, Ministry of Agriculture and Rural Affairs (Jointly built by Ministry and Province), School of Food Science and Technology, Shihezi University, Shihezi, Xinjiang 832000, China; d Texas A&M University, College Station, TX, United States; e 7 Continents Natural Products, NJ, United States

**Keywords:** Aroma, Drying method, Gray jujube powder, Harvest maturity, Quality, Vacuum freeze drying

## Abstract

This study aimed to explore the impact of different harvest maturity stages (white, semi-ripe, and fully ripe) and various drying methods [freeze drying (FD), freeze drying followed by heat pump drying, and FD followed by far-infrared drying (FD-FID)] on the quality and aroma profile of gray jujube powder. The results demonstrated that both the harvest maturity stage and drying methods significantly (*P* < 0.05) influenced the physical properties, chemical composition, and aroma profile of gray jujube powder. Notably, the combination of semi-ripe stage and FD-FID method effectively reduced browning while improving solubility, hygroscopicity, and antioxidant activity of the jujube powder, contributing to the overall enhancement of product quality. In addition, gas chromatography–mass spectrometry analysis of volatile compounds of gray jujube powder revealed differences in aroma profiles across maturity stages and drying methods. This study provides insights into how harvest timing and drying strategies can be synergistically optimized to improve the quality and aroma profile of gray jujube powder, offering a theoretical foundation and practical guidance for industrial processing.

## Highlights

• The effects of harvesting maturity coupled drying on the quality of jujube powder.
• The volatile components of gray date powder were analyzed by GC–MS.
• The effects of harvesting maturity coupled drying on the structure of jujube powder.
• The optimum preparation at semi-maturity, combined with FD-FID drying treatment.

## 1 Introduction

Gray jujube is the fruit of a plant belonging to the Rhamnaceae family, genus *Ziziphus jujuba* Mill., and is widely cultivated in subtropical and tropical regions, particularly in Australia, South Asia, East Asia, and Europe (Chen et al., 2025; Shi et al., 2022). Currently, China is the world's largest producer of jujubes, with the Xinjiang region accounting for over 40 % of the national cultivation area and contributing more than 50 % of the total output (Hou et al., 2021). Gray jujubes are renowned for their small pits and thick flesh, offering distinct significant advantages in taste and nutritional content. These fruits are rich in bioactive compounds such as vitamins, polysaccharides, cyclic adenosine monophosphate, polyphenols, and minerals. These bioactive compounds substances exhibit antioxidant, anti-inflammatory, anticancer, cardiovascular-protective, all of which play key roles properties, which are crucial in promoting human health (Liu et al., 2023, Zhu, Huang, et al., 2022, Zhu et al., 2024). However, fresh jujubes are fragile, have a short shelf life, and are prone to spoilage, making their transport difficult. Drying is widely used method to extend shelf life of fruits and vegetables, prevent spoilage and deterioration during storage and transportation. Processing jujubes into dried powder provides a practical solution for product diversification, helps expand the jujube industry supply chain, and significantly enhances the overall value of the product. This not only increases the variety of jujube-based products but also contributes to the economic sustainability of the industry (Tontul & Topuz, 2017).

Significant variations in taste and bioactive components are observed in gray jujubes at different stages of maturity. These variations are particularly evident in terms of sweetness, acidity, fiber content, polyphenols, and flavonoids (Song et al., 2019). Typically, as gray jujubes mature, their sugar content increases while their acidity decreases (Guo et al., 2015). Carbohydrates are the most abundant component in mature gray jujubes, fresh fruits containing up to 29.4 % carbohydrates (Zhao et al., 2021). Moreover, mature gray jujubes typically contain higher levels of polyphenols and flavonoids, which are beneficial for human health due to their antioxidant and anti-inflammatory properties (Shen et al., 2022). However, overripe gray jujubes can become too soft and sticky, making them unsuitable for powder processing (Yan et al., 2022). Therefore, selecting the appropriate harvest stage is essential to ensure high-quality jujube powder, as it helps retain bioactive compounds such as polyphenols, improves solubility, and reduces browning and aroma loss.

Different drying techniques can significantly affect the physicochemical properties, microstructure, nutritional components, and aroma profile of the product (Wan et al., 2025). Currently, common drying techniques used in jujube powder production include vacuum freeze-drying (FD), hot air drying (HAD), pressure difference flash drying (DIC), heat pump drying (HPD), and far infrared drying (FID). Studies have shown that FD involves two stages: the sublimation drying stage and the resolution drying stage. During the sublimation drying stage, most of the free water is removed, which significantly impacting the internal structure and texture of the dried products (Zeng et al., 2025). The resolution drying stage, on the other hand, primarily focuses on removing bound water and water that is difficult to evaporate. This stage is time-consuming and removes only a small amount of water. However, this stage can be replaced with other drying methods to significantly reduce the overall drying time (Xue et al., 2024; Yuan et al., 2022). Combining various drying technologies, especially during the drying or post-drying stages, can significantly reduce the drying time. When combined with FD technology, these methods leverage the benefits of both drying techniques (Ji et al., 2024; Liu et al., 2022). Such combined drying methods enhance the nutritional value of products, shorten drying time, save energy, and improve the overall product quality. However, studies examining how different harvesting periods and drying methods influence the nutritional quality of gray jujube powder remain limited.

Numerous studies have examined the effects of various drying methods, including DIC, FD, HPD, and FID, as well as the influence of single or combined chemical additives such as calcium stearate, magnesium stearate, and silicon dioxide, on the quality of jujube powder (Xia et al., 2023; Yang et al., 2024). However, studies focusing on how the ripeness of gray jujube at harvest, combined with different drying methods, affects both the quality and aroma profile of gray jujube powder remain limited. This study investigated how different harvesting maturity stages and drying methods influence the physicochemical properties, nutritional quality, and aroma profile of gray jujube powder. The aim was to determine the most suitable harvesting maturity stage and the optimal drying method to maximize nutrient retention in gray jujube powder and enhance aroma profile. The findings may provide scientific criteria for quality evaluation and guidelines for selecting appropriate harvesting times for gray jujube.

## 2 **Materials and** methods

### 2.1 Materials and reagents

Gray jujubes were harvested from the jujube cultivation base of the 44th Corps, Tumushuke City, China. Fresh gray jujubes at different harvest maturity stages were selected as raw materials: white jujubes (green skin), semi-ripe jujubes (green and red skin), and fully ripe jujubes (red skin) were designated as I, II, and III, respectively (Table S1). The three harvests were performed on the same three jujube trees, yielding an average of 1 kg of fruit, free from physical damage, pests, and diseases. Immediately after harvesting, the jujubes were washed with water, drained using gauze, and wiped clean. They were then quickly stored in a refrigerator at −18 °C until further experimental processing.

Food-grade enzymes (pectinase EC 3.1.1.11) and cellulase (EC 3.2.1.4) were purchased from Nanning Dongheng Huadao Biotechnology Co., Ltd. Sodium carbonate solution, aluminum nitrate, phenol, sodium hydroxide, anhydrous ethanol, methanol, n-hexane, and other analytical-grade reagents were analytical pure grade and purchased from Aladdin Reagent Company (Shanghai, China). Dichloromethane (chromatographic grade) was purchased from Xinjiang Hongdao Instrument Co., Ltd.

### 2.2 Drying methods

After cleaning and pitting, the gray jujubes were sliced into thin pieces (4.5 ± 0.5 mm) using a slicing machine. A total of 100 g of jujube slices was placed in a 1 L beaker, maintaining a material-to-liquid ratio of 1:50 (m/V, g/mL). Equal amounts of pectinase and cellulase (10,000 U/g each) were added, and the mixture was treated for 1 h at 25 °C in an ultrasonic bath operating at 40 kHz to remove soluble saccharides. The surface moisture was then removed by vacuum, and the slices were pre-frozen at −80 °C for 24 h. Then, FD, FD-HPD, and FD-FID drying treatments were applied until the moisture content (wet basis) was below 8 %. The dried samples were pulverized using an ultra-micro pulverizer for 30 s at 1200 rpm, with the pulverizing chamber temperature maintained at 20 °C. The resulting ultra-micro powdered jujube samples were sealed in vacuum bags and stored at 4 °C.

Drying process parameters (Gou et al., 2023): For FD, the cold trap temperature was set at −45 °C with a vacuum level of 0.06 kPa, and the drying duration was 24 h. Each experimental batch consisted of (100 ± 1)-g gray jujube slices, which were dried until the moisture content (wet basis) was below 5 %. For the FID method, the power was set to 8 kW, and for HPD, the temperature was maintained at 65 °C. After initial drying by either FID or HPD to a moisture ratio of 8 %, the semi-dried gray jujube slices were subsequently freeze-dried under the same FD parameters described earlier until the final moisture content (wet basis) was less than 5 %. The moisture content was determined based on the method described by (Tan et al., 2022) and calculated using the following equation:

(1) $$\mathrm{MR}=\frac{M_{t}-M_{e}}{M_{0}-M_{e}}$$

where: MR represents the moisture ratio; Me represents the moisture content of the drying time of the dried gray jujubes at a given drying time on a dry basis, kg/kg; M_t_ represents the moisture content of the dried gray jujubes at the end of a given drying time on a dry basis, kg/kg; M_0_ represents the initial moisture content of the dried gray jujubes on a dry basis, kg/kg.

### 2.3 Determination of color and degree of browning

The color difference of gray jujube powder at different harvest maturity stages was measured using a colorimeter (3nh CR-10 Plus array spectrophotometer). The brightness (L*), red-green value (a*), and yellow-blue value (b*) of gray jujube powder at different harvest maturity stages were determined. The total color difference (Δ*E*) was calculated using eq. (2). Each treatment was performed in triplicate.

(2) $$∆E=\sqrt{{\left(L^{*}-L_{0}\right)}^{2}+{\left(a^{*}-a_{0}\right)}^{2}+{\left(b^{*}-b_{0}\right)}^{2}}$$

The degree of browning (DOB) was determined following a previously reported method [21] with slight modifications. The samples were ground, and an appropriate amount of distilled water was added. After centrifugation and filtration, the supernatant was collected, and its absorbance was measured at 420 nm. If the absorbance exceeded 1.0, the sample was proportionally diluted to less than 1.0 according to the solid–liquid ratio, and the final value was expressed on a dry basis.

### 2.4 Analysis of particle size distribution, bulk density, and angle of repose of jujube powder

The particle size distribution of jujube powder was determined using a laser particle size analyzer (Winner 2005; Jinan Microparticle Instruments Co., Ltd., China). The results were expressed in terms of D10, D50, and D90 and dispersion. The dispersion was calculated using eq. (3):

(3) $$\mathrm{Span}=\frac{D_{90}-D_{10}}{D_{50}}$$

whereD10, D50, and D90 represent the particle diameters corresponding to 10 %, 50 %, and 90 % of the cumulative volume distribution, respectively.

### 2.5 Solubility and hygroscopicity measurement

Solubility was measured according to a previously reported method with slight modifications (Yang et al., 2024). A known mass of powder (m₀) was added to 10 mL of distilled water and mixed well in a water bath at 80 °C for 30 min. After centrifugation at 5000 rpm for 10 min, the supernatant was transferred to a pre-weighed aluminum dish (m1) and dried at 105 °C to a constant weight (m2). Solubility (S) was calculated using eq. (4):

(4) $$S/\%=\frac{m_{2}-m_{1}}{m_{0}}\times 100$$

The moisture absorption rate (hygroscopicity, R) was determined based on the method used by (Addo et al., 2019) with a slight modification. Accurately weighed jujube powder (m1) in a 35-mm diameter diffusion dish was placed inside a desiccator containing saturated sodium chloride solution and incubated at 25 °C. The sample mass of jujube powder (m2) was recorded at fixed intervals until the difference between two consecutive measurements was ≤1 mg, indicating moisture equilibrium. Hygroscopicity (R) was calculated using eq. (5):

(5) $$R/\%=\frac{m_{2}-m_{1}}{m_{1}}\times 100$$

### 2.6 DSC analysis

The glass transition temperature (Tg) of the samples was determined using a differential scanning calorimeter (DSC, Q200; TA Instruments, USA) following the method used by Hou et al., 2020. Approximately 8 mg of powdered sample was cooled from 20 °C to −90 °C at a rate of 20 °C/min. After equilibrating for 5 min, the sample was heated to 100 °C at the same rate. A sealed aluminum crucible was used for loading the sample, and another sealed aluminum crucible served as the blank reference during measurement.

### 2.7 Determination of total acidity and total sugars

The total acidity of jujube powder was determined by titration, following the method described by Hou et al. (2021). The sample was crushed, filtered, and diluted for pretreatment. Phenolphthalein was used as an indicator, and a 0.1 mol/L NaOH standard solution was used for titration until a light red color appeared (pH = 8.2). The total acidity was calculated as:

(6) $$\text{Total Acidity}=\frac{v\times c\times k}{m}$$

where V is the volume of alkali solution consumed, mL; C is the concentration of the alkali solution, mol/L; K is the conversion factor, and m is the sample mass (g).

The total sugar content was determined using the sulfuric acid–phenol method. The sample was ground, filtered, and diluted 103-fold. Then, 1 mL of the diluted solution was taken, mixed with 2 mL of distilled water and 1 mL of 6 % phenol solution. After thorough mixing, 6 mL of concentrated sulfuric acid was added. The mixture was vortexed and allowed to stand at 25 °C for 30 min. Absorbance was measured at a wavelength of 490 nm, using distilled water as the blank. The total sugar content was expressed as glucose equivalents. A standard curve was plotted with glucose concentration as the x-axis and absorbance as the y-axis: y = 2.2408× + 0.0479, R^2^ = 0.9995.

### 2.8 Measurement of vitamin C, flavonoids, and total phenols

The vitamin C content was determined using the 2,6-dichlorophenol titration method (Addo et al., 2020). Results were expressed in milligrams of vitamin C per gram of sample (mg/g). Briefly, 1.5 g of dried sample (or 5 g of fresh sample) was accurately weighed, and 20 mL of 2 % metaphosphoric acid was added, followed by thorough vortexing. The mixture was then centrifuged at 10,000 rpm for 15 min. Immediately afterward, 5 mL of the supernatant was transferred to a new container, and 5 mL of pH 4.0 sodium acetate buffer along with 2 mL of 2,6-dichlorophenolindophenol were added. After vortexing vigorously for 5 s, 10 mL of xylene was added, followed by another 20 s of vigorous vortexing. The mixture was then left to stand for phase separation. A standard curve was simultaneously prepared using 2,6-dichlorophenolindophenol. Finally, 3.5 mL of the xylene layer was carefully transferred to a cuvette, and absorbance was measured at 500 nm.

The flavonoid content was determined following the method reported by Lee et al. (2018). The extracted samples were mixed with Al(NO3)3 and NaOH solution, and absorbance was measured at 765 nm. The results were expressed as milligrams of rutin equivalent (RE) per gram of dry weight (mg RE/g DW). The standard curve equation using rutin as the reference standard was: y = 1440.0343× – 6.4794 (R2 = 0.9997).

Total phenol content was also determined based on the method used by Lee et al. (2018). The extracted samples were mixed with Na2CO3 and Folin–Ciocalteu reagent, and the absorbance was measured at 765 nm. The results were expressed as milligrams of gallic acid equivalent (GAE) per gram of dry weight (mg GAE/g DW). The standard curve equation using gallic acid as the standard was: y = 153.0859 × −0.9889 (R2 = 0.9962).

### 2.9 *In vitro* antioxidant assays

*In vitro* antioxidant activity was assessed based on the method described by Hou et al. (Hou et al., 2021), with slight modifications. The ABTS, DPPH, and ferric ion reducing antioxidant power (FRAP) assays were conducted as follows: A 7 mmol/L ABTS solution was mixed with a 2.45 mmol/L potassium persulfate solution at a 1:1 volume ratio and the mixture was allowed to stand at room temperature in the dark for 12–14 h. The resulting solution was diluted with 80 % methanol until the absorbance at 734 nm reached 0.70 ± 0.02. Then, 0.40 mL of Trolox standard solutions at various concentrations (0, 25, 50, 75, 100, 125, and 150 μmol/L, dissolved in 80 % methanol) was added to 3.6 mL of the ABTS solution in 10-mL test tubes. After mixing and standing for 1 min at room temperature, absorbance at 734 nm was measured. A standard curve was plotted with Trolox concentration (μmol/L) as the x-axis and absorbance as the y-axis, yielding the equation y = −0.001× + 0.3671 (correlation coefficient R^2^ = 0.9989). Sample activity was determined by diluting 0.1 mL of the extract to 0.4 mL with 80 % methanol, followed by treatment as previously described. The absorbance value was then used to calculate ABTS activity in μmol Trolox equivalents per gram of dry matter (μmol/g DM).

The DPPH radical scavenging capacity was determined as follows: A 2 mL aliquot of Trolox solutions at different concentrations (0, 20, 40, 60, 80, and 100 μmol/L, dissolved in 80 % methanol) was mixed with 4 mL of 0.1 mol/L DPPH solution (also in 80 % methanol) in 10-mL test tubes. The mixture was allowed to stand in the dark for 30 min, and the absorbance was measured at 517 nm. A standard curve was plotted with Trolox concentration (μmol/L) as the x-axis and absorbance as the y-axis, using the equation y = 0.0088× + 0.6837 (correlation coefficient R^2^ = 0.9995). A 1 mL aliquot of the extract was diluted to 2 mL with 80 % methanol and subjected to the aforementioned steps for absorbance measurement. The results were substituted into the standard curve. The DPPH radical scavenging capacity of total phenolics in the sample was expressed as μmol/g DM.

FRAP was determined as follows: A 0.2 mL aliquot of Trolox solutions at different concentrations (0, 100, 200, 300, 400, 500, 600, 700, and 800 μmol/L, dissolved in 80 % methanol) was added to 6 mL of FRAP reagent in 10-mL test tubes. After thorough mixing, the solutions were incubated at 37 °C for 30 min, cooled to room temperature, and the absorbance was measured at 593 nm. A standard curve was plotted using the equation y = 0.0012× – 0.0894 (R^2^ = 0.9943). A 0.1 mL aliquot of the extract was diluted to 0.2 mL with 80 % methanol, subjected to the aforementioned steps for absorbance measurement, and FRAP values were calculated as μmol/g DM.

### 2.10 Scanning electron microscopy analysis

The surface morphology of dried jujube powder samples was observed using a scanning electron microscope (SU5000; Hitachi, Japan). The samples were mounted on aluminum stubs and coated with a thin layer of gold. The morphological characteristics of the samples were observed at an accelerating voltage of 10 kV.

### 2.11 Analysis of volatile substance components

Headspace solid-phase microextraction was performed with slight modifications to the method described by Liu et al. (2021). Gray jujube powder of different maturities was ground into a mash, and 2 g of the sample was accurately weighed into a 20-mL extraction vial. Then, 100 μL of isoamyl phenylacetate (50 μg/mL) was added as the internal standard. The vial was placed in a 60 °C water bath and equilibrated with magnetic stirring at 250 rpm for 20 min. An extraction needle was then inserted into the vial to extract volatiles for 30 min. Prior to use, the extraction needle was activated in the gas injection port at 250 °C for 20 min.

The chromatographic separation was performed using an HP-INNOWax quartz elastic capillary column (60 m × 250 μm × 0.25 μm). The inlet and gas interface temperatures were both set to 250 °C. Helium was used as the carrier gas at a flow rate of 1.5 mL/min, with a split ratio of 4:1. The heating procedure was as follows: the initial temperature was 40 °C (held for 5 min), followed by a ramp of 5 °C/min to 250 °C, where it was maintained for 10 min. The MS conditions were as follows: ion source temperature, 230 °C; quadrupole temperature, 150 °C; EI, 70 eV; and full scan mode range, 35–550 Da.

The mass spectra of the detected compounds were analyzed using computerized matching against the NIST 14 standard spectral library, and compounds with a similarity score greater than 80 % were selected. These results were further verified through manual interpretation of the corresponding spectral data. Secondary octanol (5000 μg/kg) was used as the internal standard to quantify aroma profile using the following formula:

(7) $$\mathrm{Content of aromatic subs}\tan\mathrm{ces}\left(\mathrm{\mu g}/\mathrm{kg}\right)=\frac{\mathrm{Aroma subs}\tan\mathrm{ce}\;\mathrm{area}}{\mathrm{Internal}\;s\tan\mathrm{dard peak area}}\times \mathrm{Internal}\;s\tan\mathrm{dard concentratoin}$$

### 2.12 Statistical analysis

All data were expressed as mean ± standard deviation. Calculations were performed using Microsoft Excel 2019. One-way analysis of variance was conducted using IBM SPSS Statistics 26.0, and significant differences (*P* < 0.05) were determined using Duncan's multiple range test. Heat maps and principal component analysis were generated using Origin 21 software.

## 3 Results

### 3.1 Effects of different harvest maturity and drying methods on moisture content, color and browning degree of gray jujube powder

The moisture content, color, and browning degree are important indicators for evaluating jujube powder quality and significantly affect its stability. Table S1 shows the effects of different harvest maturity stages and drying methods on the moisture content, drying time, color, and browning degree of gray jujube powder. The results indicated that both harvest maturity and drying methods significantly influenced the moisture content, drying time, color, and browning degree of gray jujube powder (*P* < 0.05). As harvest maturity increased, the moisture content of gray jujube powder tended to decrease. This could be attributed to the higher soluble solid content in more mature gray jujubes, which facilitates faster water evaporation during drying (Shi et al., 2022). As shown in Table S1 and Fig. S2, in terms of color, the a* value of gray jujube powder decreased significantly with increasing harvest maturity (*P* < 0.05). However, the b* and L* values were significantly higher for semi-ripe jujube powder than for fully ripe jujube powder (*P* < 0.05), indicating that semi-ripe jujube powder had the most vibrant color. The total color difference (Δ*E*) was the smallest for fully ripe jujube powder and larger for semi-ripe jujube powder, indicating more pronounced color changes during drying. Although gray jujubes in the semi-ripe period exhibit a relatively stable internal structure and chemical composition that limits color changes during drying, the Δ*E* value of the resulting powder was greater, suggesting that grinding and powder formation introduced additional color variation (Dou et al., 2023). Regarding browning, semi-ripe jujube powder showed a relatively lower DOB, likely due to its lower content of reducing sugars. Reducing sugar is susceptible to the Maillard reaction at high temperatures, which leads to browning (Wang et al., 2023). Semi-ripe gray jujubes exhibited less browning during drying, which preserves the quality of jujube powder. In contrast, fully ripe jujube powder exhibited more browning due to its higher levels of reducing sugar, potentially impacting the color and flavor. Overall, the harvest maturity stage and drying methods significantly influenced the color and browning degree of gray jujube powder. Choosing the appropriate maturity stage and drying method can effectively control the color and browning degree of gray jujube powder, thus improving product quality.

### 3.2 Effects of different harvest maturity and drying methods on the particle size distribution of gray jujube powder

The particle size distribution of ultrafine gray jujube powder, as affected by different harvest maturity stages and drying methods, is shown in Table S2. Under the same drying method, gray jujube powder from stage I had a smaller particle size than that from stages II and III. Under the FD drying method, the *D*_10_, *D*_50_, and *D*_90_ values of ultrafine particles increased with harvest maturity stage (*P* < 0.05). Under the DIC-FD and FAH-FD drying methods, the particle sizes of gray jujube powder showed significant differences compared with those under the FD drying method (*P* < 0.05). Under the FD drying method, the particle dispersion significantly decreased with increasing harvest maturity (*P* < 0.05), indicating that both higher harvest maturity and combined drying methods resulted in larger particle sizes, altered physicochemical properties, reduced particle size dispersion, narrower particle size distribution, and more uniform particle size (Zhang et al., 2023). The specific surface area of the powder is closely related to its particle size: larger particle sizes correspond to smaller surface areas (Liu et al., 2022). As harvest maturity increased, the specific surface area of the powder significantly decreased (*P* < 0.05). The results indicated that under HPD-FD and FD-FID drying methods, the particle size of the powder was smaller than that obtained with FD ultrafine powder alone. Moreover, as harvest maturity increased, the particle size also gradually increased and the distribution became more uniform.

### 3.3 Effects of different harvest maturity stages and drying methods on the microstructure of gray jujube powder

**Fig. 1:**
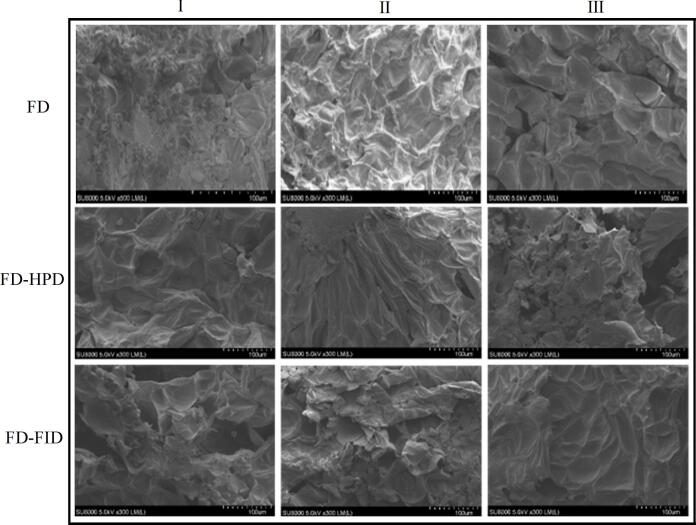
Scanning electron micrographs of gray jujube powder at different maturity stages under various drying methods.

To further investigate how harvest maturity stages combined with drying methods influence the micro-morphology of ultrafine powder, the microstructure of gray jujube powder was examined using a scanning electron microscope, as shown in Fig. 1. The results indicated that different drying methods significantly affected the powder's microstructure. Under the FD and FD-FID drying methods, the internal structure of gray jujube powder at different harvest maturity stages appeared more uniform, displaying a distinct honeycomb-like pattern and better-preserved microstructural morphology. In contrast, under the FD-HPD drying method, the gray jujube powder at the white stage exhibited a dense structure with few loose cavities, which hindered water diffusion and migration during drying and resulted in longer drying times. At the semi-ripe (half-red) stage, the tissue structure began to develop pores, which might facilitate moisture migration and thus shorten the drying time. By the fully ripe (red) stage, the tissue structure was more severely disrupted, allowing water molecules to migrate more freely from the cells and tissues compared with earlier stages. This enhanced water movement and diffusion, thereby further reducing drying time (Liu et al., 2022). From a structural and textural perspective, FD-FID appeared most effective in preserving the original microstructure of gray jujube powder. The resulting cell structure was more uniform and porous, promoting faster water absorption and penetration, which improved rehydration performance. During the semi-ripe stage, gray jujube powder retained a more intact tissue structure following FD-FID treatment.

### 3.4 Effects of different harvest maturity stages and drying methods on solubility and glass transition temperature of gray jujube powder

**Fig. 2:**
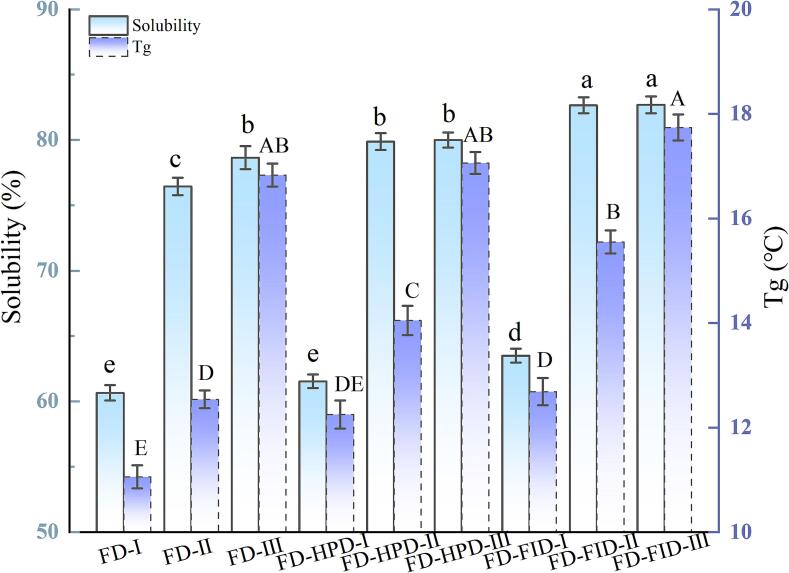
Comparison of solubility and glass transition temperature of gray jujube powder at different harvest maturity stages and under different drying methods. Note: Different uppercase and lowercase letters within the same column indicate significant differences between treatments (*P* < 0.05); the same applies to subsequent figures.

The solubility and glass transition temperature (Tg) of jujube powder are important parameters that influence its overall quality. Solubility reflects the ability of jujube powder to dissolve in water and is a key indicator for evaluating its solubility and digestibility. The Tg is closely associated with the storage stability of jujube powder. As shown in Fig. 2, both solubility and Tg of jujube powder significantly increased with increasing harvest maturity stages (*P* < 0.05). Under FD, the solubility of gray jujube powder increased significantly from 60.65 % (stage I) to 76.45 % (stage II) and 78.65 % (stage III) with the increase in the maturity stage. Similarly, Tg increased significantly from 11.06 % (stage I) to 12.54 % (stage II) and 16.83 % (stage III). When using the FD-FID combined with drying methods, the solubility and Tg of gray jujube powder increased simultaneously with harvest maturity stages. The solubility increased to 82.35 % (stage II) and 84.55 % (stage III), whereas Tg increased from 12.54 °C (stage II) and 16.83 °C (stage III) to 14.25 °C (stage II) and 18.95 °C (stage III), respectively. A similar trend was observed with the FD-HPD method. Scanning electron microscopy (SEM) analysis revealed that fully ripe jujube powder had a loose, porous structure. In contrast, samples from the white and semi-ripe stages exhibited denser structures with smaller pores. This density may explain their lower solubility (Addo et al., 2019). Additionally, gray jujube powder prepared using the FD and FD-FID drying methods exhibited a more uniform pore distribution in SEM images, facilitating rapid water absorption and diffusion, thereby improving solubility. Different drying methods had a significant influence on both solubility and Tg, likely due to moisture content, microstructure, and thermal stress. As shown in Fig. 2, for stage I powder, the solubility and Tg values were 60.65 % and 11.06 °C for FD, 61.53 % and 12.25 °C for FD-HPD, and 63.5 % and 12.69 °C for FD-FID, respectively. Similar trends were observed in stages II and III. In summary, the FID-FD drying method effectively improved both the solubility and thermal stability while preserving the microstructure of gray jujube powder. This enhancement may be attributed to the uniform porous structure formed during the FD-FID drying process, which facilitates rapid water absorption and dispersion while reducing particle agglomeration during dissolution (Addo et al., 2020).

### 3.5 Effects of different harvest maturity stages and drying methods on the hygroscopicity of gray jujube powder

**Fig. 3:**
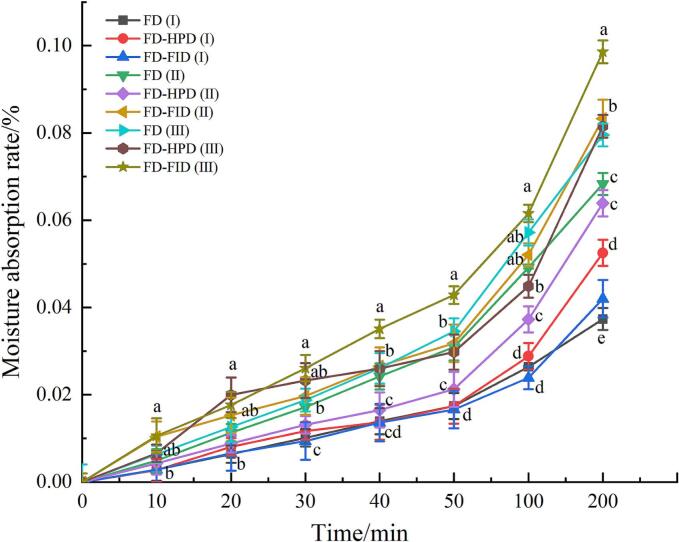
Comparison of hygroscopicity of gray jujube powder at different harvest maturity stages and under different drying methods.

Hygroscopicity research is significant for assessing the storage stability of powdered materials. Jujube powder inevitably absorbs moisture under suitable temperature and humidity conditions. Different drying methods influence hygroscopicity by altering the powder's microstructure, component retention, and glass transition temperature. Therefore, comparing the effects of drying methods on hygroscopicity is crucial for optimizing storage conditions and extending product shelf life. Fig. 3 illustrates the differences in moisture absorption rates of gray jujube powder at various harvest maturity stages after drying. After 200 min, the moisture absorption rates for gray jujube powder at the white stage, treated with FD, FD-HPD, and FD-FID drying methods, were 0.037 %, 0.053 %, and 0.042 %, respectively. In contrast, the semi-ripe stage showed rates of 0.068 %, 0.0639 %, and 0.083 %. For the fully ripe stage, the rates were 0.080 %, 0.082 %, and 0.099 %. This indicated that moisture absorption was the lowest during the white stage and the highest during the fully ripe stage. This variation may be due to changes in the pulp tissue structure of gray jujube during ripening, where greater maturity results in a more porous tissue, potentially enhancing moisture absorption (Hou et al., 2020). The more complex pore structure and network of the gray jujube powder allow water to be more easily adsorbed, impacting moisture absorption. For instance, the FD-treated gray jujube powder typically had a lower moisture absorption rate due to its porous composition. In contrast, FD-HPD and FD-FID samples exhibited relatively high moisture absorption rates, likely because high-pressure and rapid-freezing treatments altered the powder's microstructure, facilitating moisture penetration and adsorption. Additionally, structural differences influenced by maturity stages and drying methods also affect moisture absorption. Powder from less mature gray jujube, such as that at the white stage, may have a more compact structure, limiting the space available for moisture adsorption and resulting in lower moisture absorption. Conversely, powder from fully ripe stage, with looser structure, provides more sites for moisture adsorption, leading to higher moisture absorption rates. Overall, drying methods and harvest maturity stages significantly influence the moisture adsorption rate. Selecting an optimal combination can effectively reduce moisture absorption in gray jujube powder, thereby extending its shelf life.

### 3.6 Effects of different harvest maturity stages and drying methods on total acidity and total sugar content in gray jujube powder

**Fig. 4:**
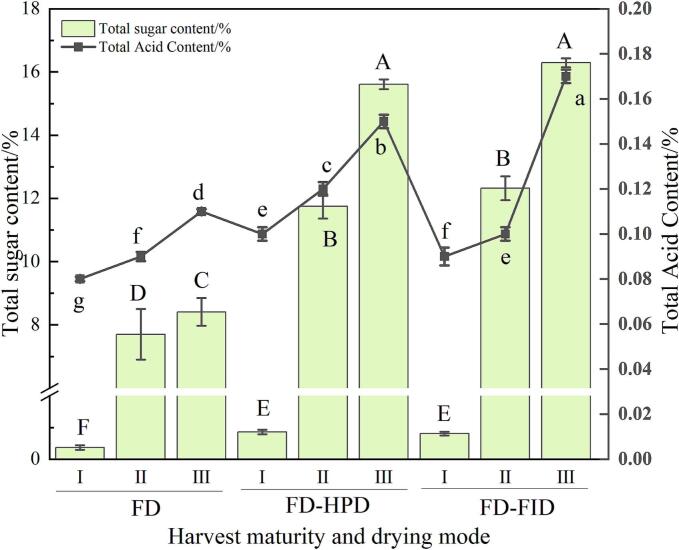
Comparison of total acidity and total sugar content in gray jujube powder under different harvest maturity stages and drying methods.

Total sugar is one of the key factors affecting the taste and quality of jujube powder. As shown in Fig. 4, the total sugar content in gray jujube powder increased with maturity. The total sugar content was lowest in jujube powder from the white stage, whereas it was highest in the fully ripe stage (*P* < 0.05). This increase may be attributed to the continuous conversion of starch into sugar during ripening, which promotes sugar accumulation. At the same harvest maturity stage, the FD-HPD and FD-FID drying methods yielded significantly higher total sugar contents compared with the FD method. This was due to more efficient water evaporation during drying, which concentrated sugars in the final product. For example, the total sugar content in gray jujube powder increased significantly as the fruit ripened. It was relatively low in the white stage (0.86 % and 0.81 %), increased considerably in the semi-ripe stage (11.75 % and 12.32 %), and reached its highest level in the fully ripe stage (15.61 % and 16.29 %. However, excessive drying temperatures may lead to Maillard and caramelization reactions, producing compounds such as furfural that can reduce the total sugar content (Zhao et al., 2021).

Total acidity is another crucial factor influencing the flavor and acceptability of dried products. As shown in the figure, the total acid content in gray jujube powder also increased with ripeness (*P* < 0.05), with the lowest value at the white stage and the highest at the fully ripe stage. This trend may be attributed to sugar metabolism during ripening, in which some sugars are converted into organic acids through fermentation, increasing total acid content (Hou et al., 2021). Drying methods also significantly affect acid retention in gray jujube powder. FD-HPD and FD-FID drying methods, which removed water more rapidly at higher temperatures compared with the traditional FD method, helped minimize acid loss and better preserve the flavor and nutritional value of jujube powder. This leads to improved taste and quality, offering consumers more desirable product options.

### 3.7 Effects of different harvest maturity stages and drying methods on total flavonoids, total phenolics, and vitamin C in gray jujube powder

**Fig. 5:**
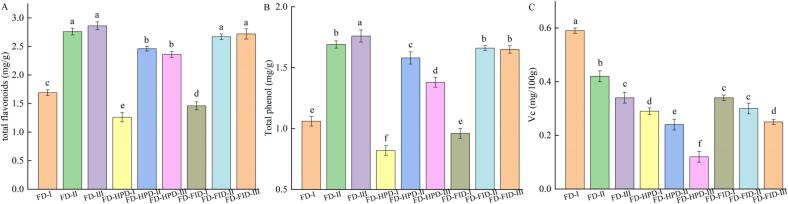
Comparison of total flavonoid content, total phenolic content, and vitamin C in gray jujube powder under different harvest maturity stages and drying methods. (A) Total flavonoids; (B) total phenols; and (C) vitamin C.

As illustrated in Fig. 5A, the total flavonoid content in gray jujube powder varied across maturity stages in the FD drying group: 1.69 mg/g in the white stage, 2.76 mg/g in the semi-ripe stage, and 2.86 mg/g in the fully ripe stage. In contrast, the FD-HPD group showed lower total flavonoid contents—1.26, 2.46, and 2.36 mg/g for the respective stages—indicating a decreasing trend with significant differences (*P* < 0.05). The FD-FID group showed flavonoid contents of 1.46, 2.67, and 2.72 mg/g for the white, semi-ripe, and fully ripe stages, respectively. Although these values were slightly lower than those in the FD group, the differences were not statistically significant (*P* > 0.05), suggesting that the FD-FID method better preserved total flavonoid content. As shown in Fig. 5B, the total phenolic content in the FD group was also significantly higher at each maturity stage: 1.06 mg/g (white), 1.69 mg/g (semi-ripe), and 1.76 mg/g (fully ripe) (P < 0.05). The FD-FID group had slightly lower levels—0.96, 1.66, and 1.65 mg/g for the corresponding stages—but the differences were not statistically significant (P > 0.05). This might be due to the lower release of polyphenol oxidase during FD-FID drying, which helped retain phenolic compounds (Mao et al., 2024). Vitamin C, a key nutrient in jujubes and an important organic acid beneficial to health, is highly susceptible to various factors such as temperature, pH, moisture, and oxygen (Wojdyło et al., 2016). As shown in Fig. 5C, the vitamin C content in the FD-treated group was 0.59, 0.42, and 0.34 mg/g for the white, semi-ripe, and fully ripe stages, respectively. These values were significantly higher than those in the FD-HPD- and FD-FID-treated groups (P < 0.05). The vacuum and oxygen-free conditions, along with low temperatures in the FD process, contributed to preserving heat-sensitive and oxidative compounds like vitamin C. During each drying process, jujube powder was inevitably exposed to oxygen; longer drying times resulted in greater vitamin C loss due to oxidation. This explains the lower vitamin C content in FD-HPD- and FD-FID-treated samples. The reduction in vitamin C during drying was primarily attributed to its heat sensitivity(Phan et al., 2021). High temperatures during drying accelerate the destruction of the molecular structure of vitamin C. At the same time, vitamin C's strong reducing properties make it highly susceptible to oxidation reactions when exposed to oxygen during drying (Yeasmin et al., 2021). In addition, the drastic changes in moisture content during drying affect the stability of vitamin C, and prolonged drying exacerbates the destructive effects of high temperatures and oxygen on vitamin C, leading to a reduction in its content (Zia & Alibas, 2021).

### 3.8 Effects of different harvest maturity stages and drying methods on the antioxidant activity of gray jujube powder

**Fig. 6:**
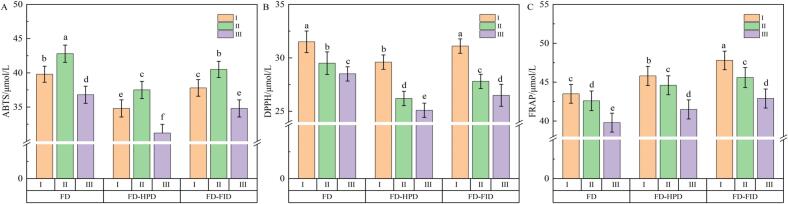
Comparison of antioxidant activity in gray jujube powder under different harvest maturity stages and drying methods. (A) ABTS free radical scavenging ability; (B) DPPH free radical scavenging ability; and (C) FRAP.

**Fig. 7:**
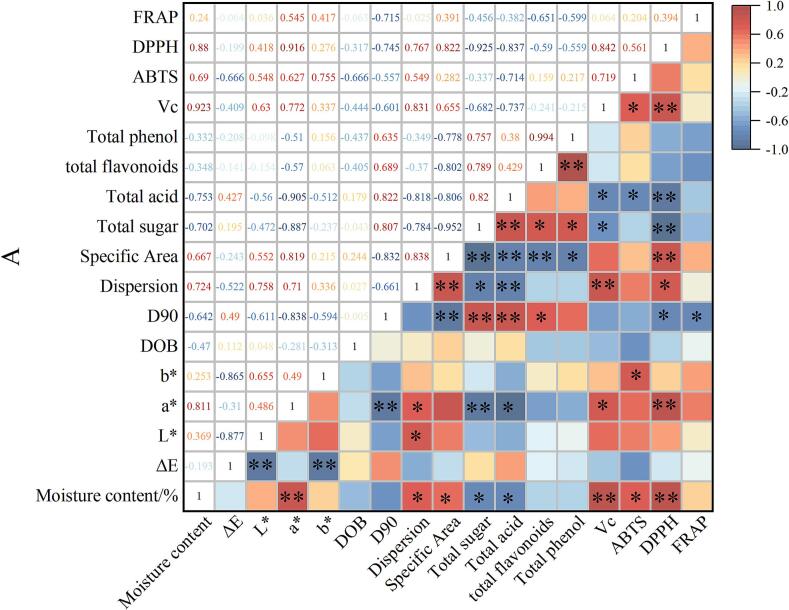
Correlation analysis of nutritional and physicochemical properties of gray jujube powder. Note: ** indicates a highly significant correlation at the 0.01 level (two-tailed); * indicates a significant correlation at the 0.05 level (two-tailed).

Clarifying the impact of harvest maturity stages and drying methods on the antioxidant activity of gray jujube powder can provide a scientific basis for optimizing the harvest time, drying parameters, and enhancing the functional value of the product. This is of practical significance for promoting the high-quality development of the jujube industry. Fig. 6A, 7B, and C illustrate the antioxidant activities of gray jujube powder harvested at different maturity stages and subjected to various drying methods. Studies have shown significant differences in antioxidant activity among gray jujubes at various maturity stages, which are closely related to the content of bioactive compounds such as total flavonoids, total phenols, and vitamin C (Chen et al., 2021). As shown in the figures, under identical drying conditions, the DPPH radical scavenging capacity and FRAP values of gray jujube powder exhibited a decreasing trend with increasing maturity (*P* < 0.05). Specifically, the ABTS radical scavenging capacities at the white, semi-ripe, and fully ripe stages were 39.8, 42.8, and 36.8 μmol/L, respectively. This indicated that the semi-ripe stage had the highest ABTS radical scavenging capacity, significantly surpassing the other maturity stages (P < 0.05). In the FD-HPD drying group, the DPPH radical scavenging capacities for gray jujube powder at the white, semi-ripe, and fully ripe stages were 40.2, 38.6, and 35.4 μmol/L, respectively. In the FD-FID drying group, the corresponding values were 41.6, 39.8, and 37.2 μmol/L, respectively. These results indicated that both FD-HPD and FD-FID drying methods slightly reduced the DPPH radical scavenging capacity of gray jujube powder compared with the FD method, although the differences were not statistically significant (*P* > 0.05). Regarding FRAP, the values measured for gray jujube powder under FD-HPD treatment were 18.5 ± 1.25 μmol/L (white maturity), 18.70 ± 1.09 μmol/L (semi-ripe), and 17.2 ± 1.15 μmol/L (fully ripe). Under the FD-FID treatment, the FRAP values were 19.2 ± 1.32 μmol/L, 19.4 ± 1.18 μmol/L, and 18.1 ± 1.21 μmol/L, respectively. Although FRAP values varied across different maturity stages, the FD-HPD and FD-FID treatments did not significantly affect FRAP values compared with the FD treatment (*P* > 0.05). In conclusion, the different harvest maturity stages and drying methods influence the antioxidant activity of gray jujube powder, and appropriate drying techniques can help preserve its antioxidant components.

### 3.9 Correlation analysis of nutritional components and physicochemical properties of gray jujube powder

The results of the correlation analysis between the nutritional components and the physicochemical properties of gray jujube powder are presented in Fig. 7. The moisture content of gray jujube powder exhibited a highly significant negative correlation (*P* < 0.01) with a*, vitamin C, and DPPH, and a significant negative correlation (*P* < 0.05) with dispersion degree, total sugar content, total acid content, specific surface area, and ABTS content. Additionally, particle size D_90_ showed a highly significant negative correlation (*P* < 0.01) with a*, specific surface area, total sugar content, and total acid content, and a significant negative correlation (*P* < 0.05) with total flavonoids, DPPH, and FRAP. Dispersion exhibited a highly significant negative correlation (*P* < 0.01) with specific surface area, total acid content, and vitamin C level, and a significant negative correlation (*P* < 0.05) with moisture content, L*, a*, total sugar content, and DPPH content. Specific surface area demonstrated a highly significant negative correlation (*P* < 0.01) with a*, D90, total sugar content, total acid content, total flavonoid content, and DPPH, and a significant negative correlation (*P* < 0.05) with moisture content and total phenols. Among the nutritional components, total phenol content showed significant positive correlations (*P* < 0.05) with total flavonoids, ABTS, and FRAP, indicating that total phenols may play a key role in antioxidant activity. Furthermore, the positive correlation (*P* < 0.05) between total acid and total sugar contents may reflect the balance between acids and sugars in gray jujube powder. These correlation results help clarify the interactions among various components in gray jujube powder and provide theoretical support for quality control and the optimization of its functional properties (Zhu, Kou, et al., 2022). The findings indicate that complex interrelationships exist between the nutritional components and physicochemical characteristics of gray jujube powder, which are important for guiding its processing and application.

### 3.10 Effects of different harvest maturity stages and drying methods on the aroma profile of gray jujube powder

Different harvest maturity stages and drying methods significantly impacted the types, contents, and volatile composition of the aroma profiles in gray jujube powder. These factors influenced the aroma by altering endogenous enzyme activity during the fruit maturation stage and the thermochemical reaction pathways during drying, resulting in distinct aroma profiles. As shown in Table S3, gray jujube powder samples from different harvest maturity stages and drying methods contained various volatile components, including esters, aldehydes, acids, alcohols, ketones, and alkanes. Among these, ester compounds were the most abundant and typically exhibited fruity or floral characteristics, contributing prominently to the characteristic aroma of jujube powder. Although aldehydes are less abundant, they play a significant role in imparting fresh, fruity aromas. Acids and alcohols provide the powder with its basic sourness and alcoholic aroma, enhancing the overall aroma profile (Pan et al., 2022). Additionally, although ketones and alkanes are less abundant than esters and aldehydes, they still contribute to the aroma profile of gray jujube powder (Liu et al., 2024). Ketones typically exhibit sweet or floral aromas, while alkanes help provide a stable foundation for the aroma (Jia et al., 2024). Under different drying methods, gray jujube powder at the white stage contained unique alcohol compounds such as isopropyl alcohol and decanol, as well as linolenic acid. In contrast, powder from the semi-ripe and fully ripe stages contained unique compounds including benzaldehyde, methyl 2-methylbutyrate, methyl cinnamate, 3-methyl-2-butanone, 2-nonanone, 2-methyl-3-octanone, and methyl isovalerate. These compounds are especially characteristic of fully ripe fruit. GC–MS analysis clearly identified these volatiles, enhancing understanding of how harvest maturity stages and drying methods affect the aroma profile. Drying methods play a key role in regulating the retention and transformation of volatile components. Vacuum FD helps retain natural esters and alcohols in ripe fruit to a greater extent. These changes are driven by synergistic effects between fruit maturity and the drying methods. By precisely controlling processing parameters, the flavor profile of gray jujube powder can be optimized. For applications requiring a high ester content, using fully ripe fruits in combination with the FD method is ideal (Cai et al., 2024).

In summary, different harvest maturity stages and drying methods significantly affected the volatile components of gray jujube powder. By optimizing drying conditions, it is possible to regulate the production and proportions of these volatile compounds, thereby improving the aroma profiles of gray jujube powder.

**Fig. 8:**
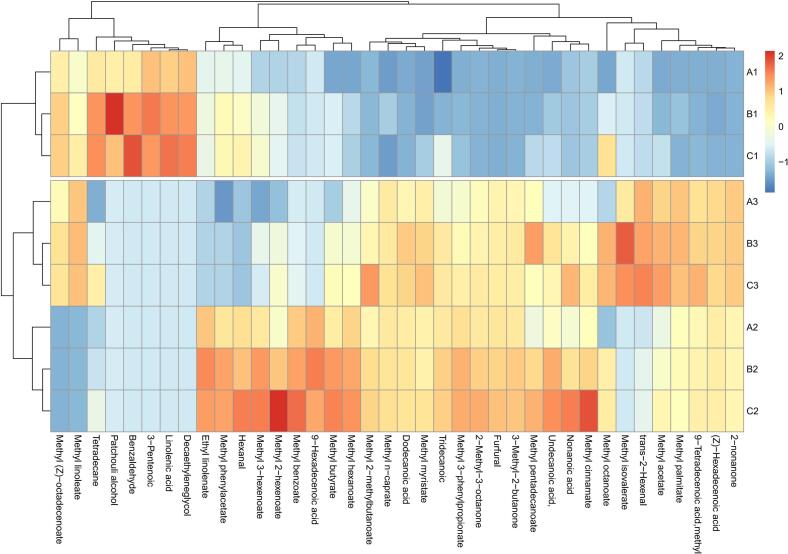
Heatmap of VOCs in gray jujube powder across different harvest maturity stages and drying methods. Note: A1, B1, and C1 indicate FD-I, FD-II, and FD-III, respectively; A2, B2, and C2 indicate FD-HPD-I, FD-HPD-II, and FD-HPD-III, respectively; A3, B3, and C3 indicate FD-FID-I, FD-FID-II, and FD-FID-III, respectively.

Fig. 8 illustrates the thermogram of volatile organic compounds (VOCs) in gray jujube powder under various harvest maturity stages and drying methods, showing the distribution of VOCs under different treatment conditions. The thermogram reveals that both ripeness and drying treatments significantly affected the aroma profile of gray jujube powder. The types and quantities of VOCs present at the white, semi-ripe, and fully ripe stages varied after drying, potentially due to biochemical changes occurring in gray jujubes at different maturity stages. Specifically, gray jujube powder from the white stage showed fewer types of VOCs after FD, although the concentrations of certain individual compounds were higher, possibly due to the accumulation of sugars and acids in gray jujubes (Jia et al., 2024). In contrast, powder from the semi-ripe and fully ripe stages exhibited a more intricate VOC profile, likely resulting from the more complete transformation of aroma precursors with increasing ripeness (Song et al., 2019). Additionally, the drying method significantly influenced the VOC composition of gray jujube powder. Both FD-HPD and FD-FID treatments produced distinct VOC profiles across the different maturity stages. FD-HPD tended to better preserve volatile aroma profile, whereas FD-FID might have promoted the production or transformation of certain aroma profile. These variations may be attributed to the temperature and humidity regulation during drying, affecting enzyme activity and thermochemical reaction pathways in gray jujubes (Yang et al., 2024). In summary, the aroma profile of gray jujube powder was influenced by both harvest maturity stage and drying method. By optimizing both the harvesting time and the drying process, the aroma profile of gray jujube powder can be effectively regulated, thereby enhancing its antioxidant activity and overall quality.

## 4 Conclusions

This study systematically investigated the effects of different harvest maturity stages and drying methods on the quality and aroma of gray jujube powder. The findings demonstrated that both factors significantly influenced various characteristics of the powder, including hardness, brittleness, total acidity, total sugar content, moisture content, drying time, color, browning degree, hygroscopicity, antioxidant activity, nutritional composition, and volatile compound profile. Notably, jujubes harvested at the semi-ripe stage and processed using the FD-FID drying method exhibited improved hardness and brittleness, reduced moisture content, enhanced color, decreased browning, and increased hygroscopicity and antioxidant activity, thereby promoting anti-caking properties of the powder. Furthermore, the aroma profile of gray jujube powder was significantly shaped by both harvest maturity stages and drying methods. By selecting appropriate maturity stages and optimizing drying conditions, the generation and composition of volatile compounds could be regulated, thereby enhancing the powder's aromatic quality. Overall, this study provides valuable insights for improving the processing and quality of gray jujube powder.

## CRediT authorship contribution statement

**Zhanxia Liu:** Writing – original draft, Investigation. **Hongbin Wu:** Writing – review & editing. **Yinglin Du:** Investigation. **Xinwen Jin:** Validation. **Tarun Belwal:** Writing – review & editing. **Hui Yang:** Supervision. **Xizhe Fu:** Writing – review & editing, Supervision, Conceptualization.

## Declaration of competing interest

The authors declare that they have no known competing financial interests or personal relationships that could have appeared to influence the work reported in this paper.

## Data Availability

Data will be made available on request.

## Appendix A Supplementary data

Supplementary material Image 1
