# Supplementary material for: Effects of harvest maturity coupled with various drying methods on the quality and aroma composition of gray jujube powder
Source: Food Chem X. 2025 Aug 20;30:102916. doi: 10.1016/j.fochx.2025.102916 (PMC12398869; doi:10.1016/j.fochx.2025.102916)
Supplement: Supplementary file 1 — Supplementary material [file mmc1.docx]

**Supplemental Figure 1**


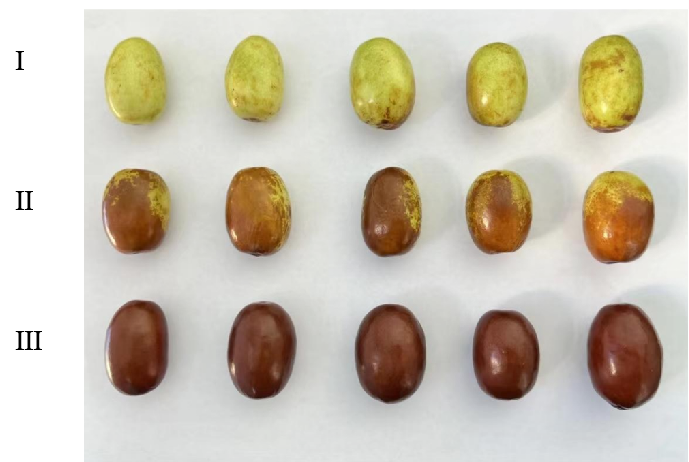


**Figure S1. Different harvest maturity of gray jujube fruit**

**Supplemental Figure 2**


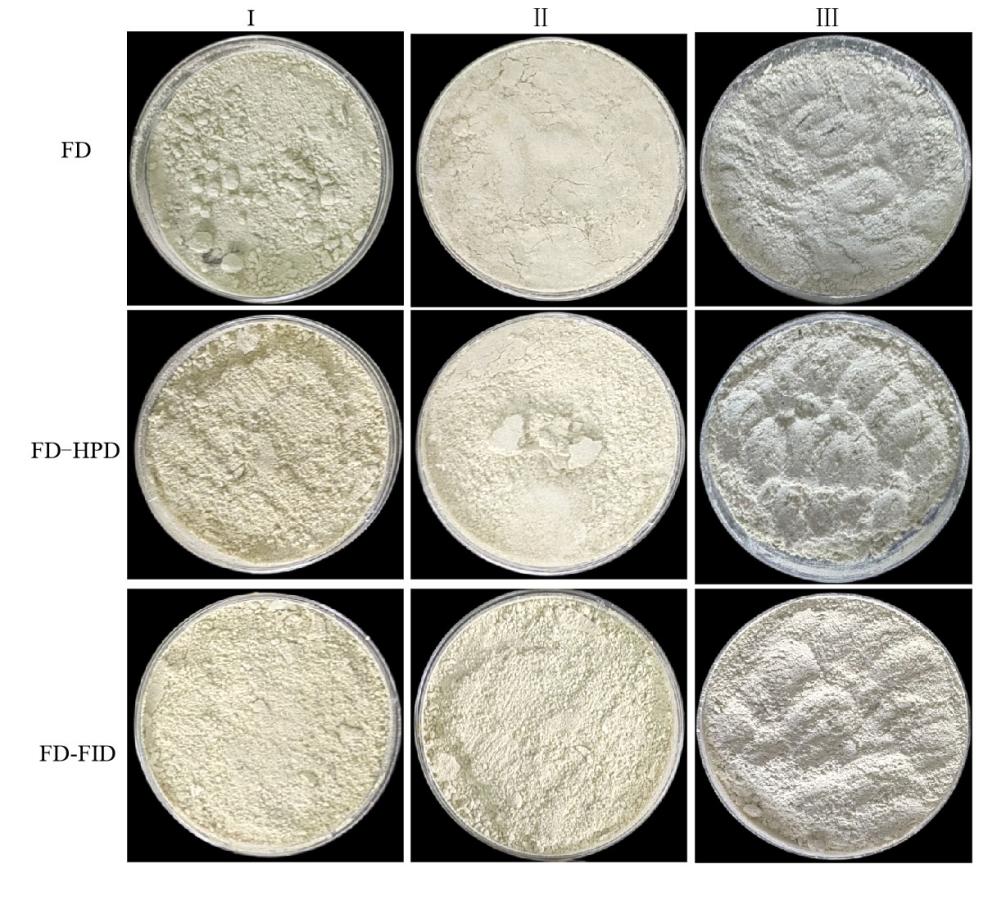


**Figure S2. Gray jujube powder from different harvest maturity and drying methods**

**Supplemental Figure 3**


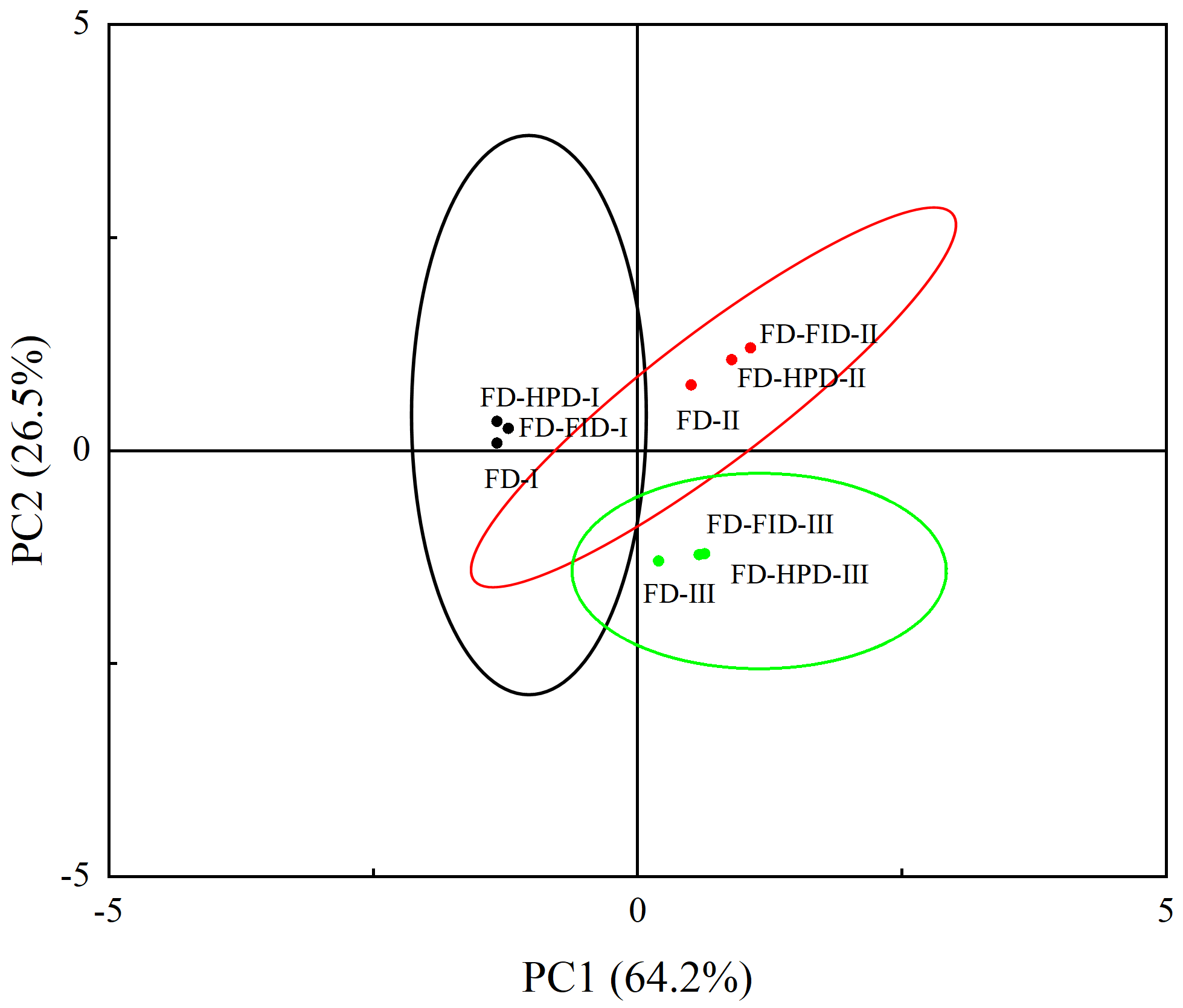


**Figure S3. PCA score plot of VOCs in dried jujube powder affected by different harvest maturity and drying methods**

**Supplemental Table 1**

**Table S1 Effects of Different Harvest Maturity and Drying Methods on Moisture Content, Drying Time, Color, and Browning Degree of Grey Jujube Powder**

| Drying method | Maturity | Water content/% | Δ*E* | *L** | *a** | *b** | DOB/（Abs/g.d.） |
| --- | --- | --- | --- | --- | --- | --- | --- |
| FD | I | 7.15±0.25^a^ | 35.88±1.25^e^ | 44.21±1.46^a^ | 21.08±1.81^a^ | 19.17±1.81^b^ | 0.26±0.01^c^ |
|  | Ⅱ | 5.85±0.17^b^ | 37.83±1.64^d^ | 42.45±1.80^c^ | 18.05±1.33^b^ | 23.33±1.33^e^ | 0.23±0.02^d^ |
|  | Ⅲ | 4.51±0.25^c^ | 31.07±1.31^f^ | 39.65±1.42^d^ | 17.65±2.10^c^ | 18.26±2.10^f^ | 0.34±0.01^b^ |
| FD-HPD | I | 4.25±0.22^c^ | 35.43±1.33^e^ | 42.52±1.81^c^ | 18.44±1.31^b^ | 19.57±1.31^e^ | 0.34±0.02^b^ |
|  | Ⅱ | 3.79±0.13^e^ | 43.41±1.25^a^ | 43.47±1.76^b^ | 17.39±1.96^c^ | 24.64±1.56^a^ | 0.31±0.01^bc^ |
|  | Ⅲ | 3.55±0.12^f^ | 31.34±1.26^f^ | 34.09±1.21^g^ | 13.45±1.42^e^ | 13.58±1.30^g^ | 0.41±0.01^a^ |
| FD-FID | I | 5.99±0.15^b^ | 38.95±1.09^c^ | 36.04±1.55^f^ | 21.28±1.21^a^ | 20.32±1.40^d^ | 0.30±0.02^bc^ |
|  | Ⅱ | 4.81±0.22^c^ | 40.71±1.52^b^ | 38.38±1.03^e^ | 18.00±1.63^b^ | 21.24±2.63^c^ | 0.27±0.01^c^ |
|  | Ⅲ | 4.06±0.21^d^ | 37.11±1.22^d^ | 36.97±1.44^f^ | 14.39±2.09^d^ | 17.75±2.69^f^ | 0.36±0.02^b^ |

Note: Different lowercase letters in the same column indicate significant differences in treatments (*P*<0.05), the same as follows.

**Supplemental Table 2**

**Table S2 The Effect of Drying Methods on the Physical Properties of Gray jujube Powder**

| Drying method | Maturity | D_10_/μm | D_50_/μm | D_90_/μm | Discrepancy | Specific surface area/（m^2^/kg） |
| --- | --- | --- | --- | --- | --- | --- |
| FD | I | 7.81±0.25^i^ | 27.45±2.10^d^ | 68.55±2.10^e^ | 3.36±0.07^a^ | 327.45±4.22^a^ |
|  | Ⅱ | 8.30±0.17^f^ | 32.30±2.25^c^ | 72.30±2.05^c^ | 3.24±0.01^b^ | 303.21±4.17^c^ |
|  | Ⅲ | 9.22±0.19^c^ | 39.22±2.38^b^ | 81.87±2.35^b^ | 3.11±0.01^c^ | 296.66±5.10^d^ |
| FD-HPD | I | 7.95±0.15^h^ | 28.05±2.17^d^ | 69.36±2.10^d^ | 3.15±0.03^c^ | 320.21±3.67^a^ |
|  | Ⅱ | 8.46±0.11^e^ | 33.95±2.10^c^ | 73.12±2.17^c^ | 3.00±0.05^d^ | 298.66±4.35^d^ |
|  | Ⅲ | 9.67±0.15^b^ | 41.23±2.32^a^ | 82.33±2.20^a^ | 2.86±0.04^f^ | 290.12±3.75^d^ |
| FD-FID | I | 8.03±0.17^g^ | 28.22±2.13^d^ | 7-±2.17^d^ | 3.02±0.02^d^ | 315.26±4.08^b^ |
|  | Ⅱ | 8.66±0.12^d^ | 33.99±2.20^c^ | 74.05±2.10^c^ | 2.90±0.03^e^ | 289.00±3.35^e^ |
|  | Ⅲ | 9.82±0.15^a^ | 41.87±2.08^a^ | 82.37±2.08^a^ | 2.76±0.02^g^ | 275.36±5.00f |

**Supplemental Table 3**

**Table S3 Volatile substances in dried jujube powder at different maturity levels and drying methods**

| Number | Volatile substances |  | Relative Content/ % | | | | | | | | |
| --- | --- | --- | --- | --- | --- | --- | --- | --- | --- | --- | --- |
|  |  |  | FD | | | FD-HPD | | | FD-FID | | |
|  |  | RI | I | Ⅱ | Ⅲ | I | Ⅱ | Ⅲ | I | Ⅱ | Ⅲ |
|  | **Esters** | |  | | | | | | | | |
| 1 | Methyl caproate | 1174.7 | 1.08 | 0.70 | 0.01 | 1.05 | 0.71 | 0.84 | 1.17 | 0.75 | 0.85 |
| 2 | Methyl butyrate | 969.5 | 0.20 | 0.21 | - | 0.27 | 0.24 | 0.16 | 0.27 | 0.25 | 0.17 |
| 3 | Methyl 2-methylbutyrate | 995.0 | - | 0.09 | - | - | 0.12 | 0.08 | - | 0.12 | 0.14 |
| 4 | Methyl isovalerate | 1005.7 | - | - | - | - | - | 0.18 | - | - | 0.15 |
| 5 | Methyl hexanoate | 1071.8 | 2.93 | 2.67 | 0.02 | 3.31 | 2.76 | 1.96 | 3.16 | 2.78 | 2.03 |
| 6 | Methyl hex-3-enoate | 1246.2 | 0.24 | 0.21 | - | 0.31 | 0.24 | 0.14 | 0.32 | 0.25 | 0.12 |
| 7 | Methyl 2-hexenoate | 1215.5 | 0.77 | 0.47 | - | 0.83 | 0.54 | 0.41 | 0.80 | 0.64 | 0.42 |
| 8 | Methyl octanoate | 1379.6 | 0.58 | 0.30 | - | 0.63 | 0.37 | 0.39 | 0.77 | 0.37 | 0.38 |
| 9 | Methyln-nonanoate | 1481.8 | - | 0.06 | - | - | 0.10 | 0.08 | - | 0.15 | 0.11 |
| 10 | Methyl n-caprate | 1587.5 | 19.20 | 15.78 | 0.15 | 20.55 | 15.40 | 14.35 | 17.76 | 15.31 | 14.35 |
| 11 | Methyl benzoate | 1618.0 | 1.07 | 2.38 | 0.01 | 1.22 | 2.59 | 0.84 | 1.26 | 2.91 | 0.86 |
| 12 | Methyl undecanoate | 1689.3 | 0.35 | 0.29 | - | 0.38 | 0.37 | 0.31 | 0.42 | 0.38 | 0.28 |
| 13 | Methyl phenylacetate | 1750.5 | 1.65 | 1.00 | 0.01 | 1.76 | 1.09 | 0.63 | 1.77 | 1.07 | 0.62 |
| 14 | Methyl dodecanoate | 1796.8 | 26.95 | 27.45 | 0.26 | 26.22 | 27.31 | 28.00 | 25.87 | 26.99 | 26.90 |
| 15 | Methyl 3-phenylpropionate | 1831.3 | 0.47 | 1.21 | 0.01 | 0.53 | 1.34 | 0.88 | 0.62 | 1.33 | 0.94 |
| 16 | Methyl myristate | 2041.8 | 4.99 | 5.14 | 0.05 | 4.64 | 4.94 | 5.28 | 5.82 | 5.22 | 5.43 |
| 17 | 9-Tetradecenoic acid,methyl | 2079.0 | 19.54 | 21.45 | 0.24 | 18.73 | 21.19 | 23.58 | 19.49 | 21.31 | 24.91 |
| 18 | Methyl cinnamate | 2084.1 | - | 0.08 | - | - | 0.12 | 0.07 | - | 0.18 | 0.09 |
| 19 | Methyl pentadecanoate | 2105.4 | 0.11 | 0.11 | - | 0.10 | 0.16 | 0.17 | 0.15 | 0.17 | 0.11 |
| 20 | Methyl palmitate | 2210.6 | 2.38 | 2.27 | 0.03 | 2.48 | 2.23 | 2.75 | 2.25 | 2.15 | 2.64 |
| 21 | (Z)-Hexadecenoic acid | 2297.0 | 12.87 | 14.38 | 0.16 | 11.59 | 13.97 | 14.83 | 12.38 | 13.48 | 14.45 |
| 22 | Methyl (Z)-octadecenoate | 2440.0 | 0.36 | - | - | 0.42 | - | 0.18 | 0.42 | - | 0.18 |
| 23 | Methyl linoleate | 2488.2 | 0.86 | - | 0.01 | 0.89 | - | 0.72 | 1.13 | - | 0.71 |
| 24 | Ethyl linolenate | 2555.9 | 0.31 | 0.55 | - | 0.38 | 0.67 | - | 0.33 | 0.61 | - |
|  | Total | - | 96.91 | 96.78 | 0.97 | 96.29 | 96.47 | 96.83 | 96.15 | 96.40 | 96.84 |
|  | **Aldehydes** | |  | | | | | | | | |
| 25 | Hexanal | 1069.8 | 0.12 | 0.16 | - | 0.20 | 0.18 | - | 0.24 | 0.22 | - |
| 26 | trans-2-Hexenal | 1215.5 | 0.22 | 0.15 | 0.01 | 0.22 | 0.21 | 0.62 | 0.22 | 0.21 | 0.67 |
| 27 | Benzaldehyde | 960.7 | 0.12 | - | - | 0.20 | - | - | 0.24 | - | - |
| 28 | Furfural | 1156.2 | - | 0.83 | 0.01 | - | 0.89 | 0.69 | - | 0.94 | 0.70 |
|  | Total |  | 0.46 | 1.15 | 0.01 | 0.62 | 1.28 | 1.32 | 0.70 | 1.36 | 1.36 |
|  | **Acids** | |  | | | | | | | | |
| 29 | 3-Pentenoic | 2336.5 | 0.71 | - | - | 0.88 | - | - | 0.79 | - | - |
| 30 | Tridecanoic | 2884.7 | 0.14 | 0.11 | - | 0.15 | 0.11 | 0.10 | 0.18 | 0.11 | 0.10 |
| 31 | Linolenic acid | 2451.5 | 0.11 | - | - | 0.13 | - | - | 0.14 | - | - |
| 32 | 9-Hexadecenoic acid | 1941.8 | - | 0.20 | - | - | 0.23 | - | - | 0.19 | - |
|  | Total | - | 0.96 | 0.31 | - | 1.16 | 0.34 | 0.10 | 1.11 | 0.30 | 0.10 |
|  | Alkane | |  |  |  |  |  |  |  |  |  |
| 33 | Patchouli alcohol | 2163.7 | 0.08 | 0.03 | - | 0.09 | 0.03 | 0.03 | 0.09 | 0.03 | 0.03 |
|  | **Alcohols** | |  |  |  |  |  |  |  |  |  |
| 34 | linalool | 1635.5 | 0.06 | - | - | 0.14 | - | - | 0.09 | - | - |
| 35 | Decaethylene glycol | 1535.4 | 1.54 | - | - | 1.70 | - | - | 1.87 | - | - |
|  | Total | - | - | 0.95 | 0.01 | - | 1.07 | 0.84 | - | 1.07 | 0.78 |
|  | **Ketones** | | - | 0.52 | 0.01 | - | 0.50 | 0.67 | - | 0.49 | 0.65 |
| 36 | 3-Methyl-2-butanone | 2456.5 | - | 0.27 | - | - | 0.30 | 0.22 | - | 0.34 | 0.23 |
| 37 | 2-nonanone | 2909.8 | 0.06 | - | - | 0.14 | - | - | 0.09 | - | - |
| 38 | 2-Methyl-3-octanone | 3075.8 | 1.54 | - | - | 1.70 | - | - | 1.87 | - | - |
|  | Total | - | 1.60 | 1.74 | 0.02 | 1.84 | 1.87 | 1.72 | 1.96 | 1.90 | 1.67 |

-- Indicates not detected
